# Numbness and Weakness Recovered at a Less Extent in Patients with Lumbar Disc Herniation after Percutaneous Endoscopic Lumbar Discectomy

**DOI:** 10.1155/2019/4642701

**Published:** 2019-12-23

**Authors:** Yuming Wang, Fuqiang Gao, Haibo Zou

**Affiliations:** Department of Orthopedics, China-Japan Friendship Hospital, 100029 Beijing, China

## Abstract

**Background:**

Patients with lumbar disc herniation (LDH) may present with motor disorders and various sensory disorders, among which pain and numbness are the most common ones. Percutaneous endoscopic lumbar discectomy (PELD) is reported to be both safe and effective. However, most of the previous studies focused on the recovery of pain, and the relief extent of numbness and weakness has rarely been reported. The Sciatica Bothersomeness Index (SBI) is a self-assessment tool for LDH patients. It has demonstrated acceptable reliability, construct validity, and responsiveness.

**Objectives:**

Our aim was to explore the curative effect of percutaneous endoscopic lumbar discectomy and to compare the various extent of relief among pain, numbness, and weakness.

**Methods:**

The medical records of patients admitted for LDH from September 2016 to December 2018 were collected, and the patients were followed up for 3 months to evaluate the relief of their clinical symptoms. Preoperative and postoperative total scores and subitem scores of SBI were compared to evaluate the relief of pain, numbness, and weakness. Surgical outcomes of PELD were evaluated by the Nakai score, and patients were divided into two groups accordingly, which were the relief group (excellent and good in the Nakai score) and the less relief group (fair and poor in the Nakai score). Risk factors for PELD outcomes and preoperative presence of numbness and/or weakness were analyzed by the logistic model, and *p* value less than 0.05 was considered significant.

**Results:**

A total of 86 patients met the inclusion criteria and acquired 3 months follow-up. Relief extent of pain, numbness, and weakness, was 82%, 41%, and 21%, respectively. There were 71 cases in the relief group and 15 cases in the less relief group. Results of the logistic regression analysis showed that the preoperative pain score of SBI (*p*=0.002; OR: 1.647 (1.199–2.261)) was a relatively independent risk factor for PELD outcomes, and multiplicativity of duration of preoperative symptoms and imaging grade [*p*=0.004; OR: 1.015 (1.005–1.026)] was a relatively independent risk factor for preoperative presence of numbness and/or weakness.

**Conclusions:**

PELD had a good curative effect in the treatment of LDH. Patients of LDH recovered best from pain, followed by numbness and weakness after PELD. Higher level of patients self-reported preoperative pain indicated a better surgical outcome for LDH patients, and preoperative long duration of symptoms together with a severe compression of nerve root significantly increased the risk of presenting numbness and/or weakness.

## 1. Introduction

Patients with lumbar disc herniation (LDH) usually present with symptoms like pain, numbness, and weakness, which have a negative influence on their social functions [1, 2]. Percutaneous endoscopic lumbar discectomy (PELD) first appeared in 1992 [3] and has been widely used in clinical practice for lumbar spine diseases due to less invasiveness and faster recovery compared with traditional surgery [4–6]. Many studies have reported that PELD could achieve comparative therapeutic effect as traditional surgery. However, most previous studies applied the Japanese Orthopaedic Association (JOA) score, the Visual Analog Scale for Pain (VAS Pain), the Oswestry Disability Index (ODI) score, or the 36-Item Short-Form Health Survey score (SF36) to evaluate surgery outcomes, which mainly provided evidence about patients' recovery of either overall function of neurodeficit, pain, social dysfunction, or quality of life [7–9], while variable relief extent of symptoms such as numbness and weakness has rarely been reported [10].

Sciatica Bothersomeness Index (SBI) is a self-assessment tool to assess the severity of sciatica. It contains four items, which can evaluate the main symptoms of LDH patients quantitatively, such as pain, numbness, and weakness [11]. It has been applied in previous studies about lumbar disc herniation and has demonstrated acceptable reliability, construct validity, and responsiveness [12, 13]. The purpose of this study was to investigate the curative effect of endoscopic treatment of lumbar disc herniation. The SBI score was used both preoperatively and postoperatively to evaluate the variable extent of relief among pain, numbness, and weakness. We hypothesized that numbness and weakness recovered at a less extent than pain in patients with lumbar disc herniation after percutaneous endoscopic lumbar discectomy.

## 2. Methods

### 2.1. Participants

LDH patients admitted for PELD from September 2016 to December 2018 were enrolled in this study. The criteria for inclusion were as follows: (1) complained of unilateral lower limb radiating pain, numbness, weakness, or other symptoms caused by single nerve root compression; (2) with symptoms consistent with preoperative imaging; and (3) with no significant relief of symptoms after 6 weeks of regular conservative treatment. The exclusion criteria were as follows: (1) with lumbar spinal stenosis; (2) and/or with lumbar instability; and (3) with spondylolisthesis or deformity.

### 2.2. Surgical Procedure

Two approaches for PELD were selected according to disk segments of herniation, which were transforaminal approaches for L45 and interlaminar approaches for L5S1. Surgical procedure was performed by one doctor under local anesthesia with 1% lidocaine, and the patients were laid in the prone position. A cannula was inserted into the spinal canal under the guidance of the C-arm X-ray machine. Free disk fragments were removed as much as possible. Microscopically, free movement of the nerve root and dural sac with the change of abdominal cavity pressure during breathing indicated that the goal of nerve decompression had been achieved.

### 2.3. Postoperative Management

All patients were treated with neurotrophic drugs routinely after operation and were able to get out of bed with a brace 24 hours after operation. After 3 weeks postoperation, the patients were instructed to return to everyday activities gradually.

### 2.4. Outcome Measurement

(1) Demographic data included gender, age, and body mass index (BMI); (2) clinical data included duration of preoperative symptoms, the SBI scores for evaluating preoperative symptoms, and the ODI score for evaluating social dysfunction. The SBI score is a patient self-rated instrument usually applied to evaluate the severity of sciatica, which contains 4 items and includes the most common symptoms of LDH patients, such as pain, numbness, and weakness. Each item has a range of score from 0 to 6. The index has labels at the categories 0 (not bothersome), 3 (somewhat bothersome), and 6 (extremely bothersome), which provides a total score from 0 to 24 when summing up the ratings across the 4 items [11]. (3) Imaging data were graded by the Pfirrmann grading system in which nerve root compression was graded into three categories based on preoperative MR images (A: normal or contact; B: deviation; and C: compression) [14]. Evaluation of the image data was completed independently by two attending doctors who had been specializing in spine surgery for at least five years. Any discordance in the evaluation of the image results was discussed, and final agreement was reached before they were recorded for analysis. (4) Follow-up data: all patients were followed up at 3 months after operation. The relief of symptoms was evaluated by the change of the total SBI score and each subitem score. The extent of symptom relief was calculated by the formula (preoperative scores−postoperative scores)/preoperative scores. The curative effect of PELD was evaluated by the Nakai score (Table 1) [15].

### 2.5. Statistical Analysis

All data were analyzed using the Statistical Package for the Social Sciences (SPSS) version 22 (IBM, USA). For all qualitative variables, the total number of patients and the percentage were provided. For quantitative variables, the mean and standard deviation were provided. To reveal the relationship between social dysfunction and different symptoms like pain, numbness, and weakness, correlations between preoperative ODI and the SBI score and its subitem scores were analyzed by the Pearson correlation analysis. The risk factors for curative effect and preoperative presence of numbness and/or weakness were analyzed by the logistic regression model. Factors according to its clinical significance were first assessed individually as predictive variables by the logistic regression analysis, and the final model included all the predictive variables with *p* values less than 0.2 as covariates, which were further assessed together by the logistic regression. *p* value less than 0.05 was taken as the criterion for covariates being significant risk factors.

## 3. Results

A total of 87 patients were enrolled in this study, and one case was lost to follow-up. The 86 enrolled patients included 44 males and 42 females, aged from 21–80, with an average age of 49. Demographic and clinical characteristics of the study patients are listed in Table 2.

**Figure 1:**
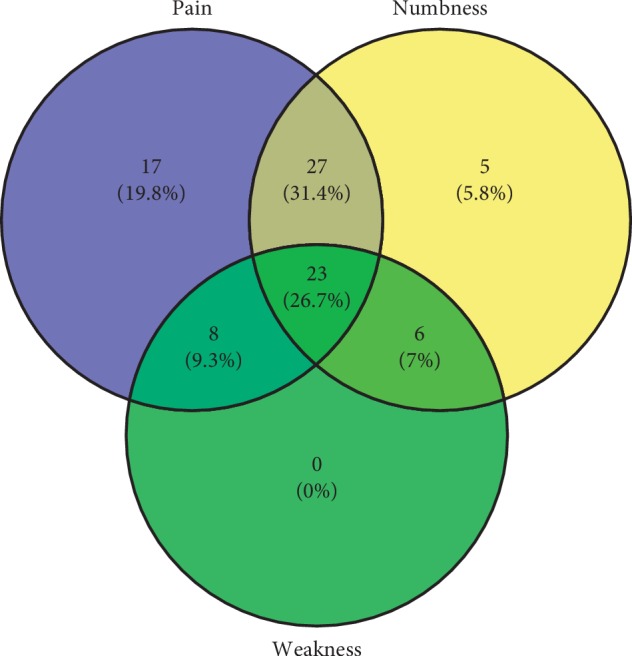
Proportions of various symptoms.

Proportions of pain, numbness, and weakness are shown in Figure 1. Pain was the most common symptom, followed by numbness and weakness. Before surgery, 75 (87.2%) out of 86 patients had pain, 61 patients (70.9%) had numbness, and only 37 patients (43%) had weakness. Only 23 patients (26.7%) had all these 3 symptoms at baseline.

Results of the Pearson correlation analysis showed that the ODI score displayed a positive correlation with the preoperative SBI total score, pain score, and weakness score, while no significant correlations were found between the ODI score and the numbness score (Table 3).

**Figure 2:**
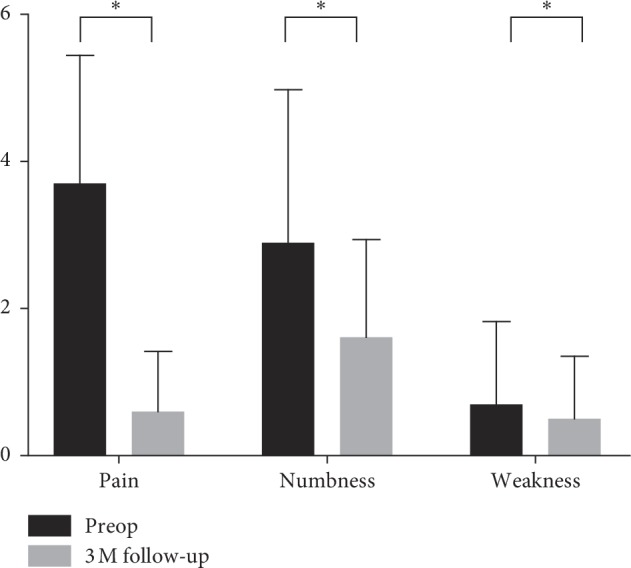
Relief extent of various symptoms at 3 months follow-up. ^*∗*^Indicates *p* < 0.05.

At 3 months after operation, the mean ODI score of the study population was decreased to 16.2 (11.33), *p* < 0.001. The mean SBI total score was decreased to 3.2 (2.32), *p* < 0.001, with a symptom relief rate of 65%. The mean pain score was decreased to 0.6 (0.82), *p* < 0.001, with a symptom relief rate of 82%. The mean numbness score was decreased to 1.6 (1.34), *p* < 0.001, with a symptom relief rate of 41%. The mean weakness score was decreased to 0.5 (0.85), *p* < 0.001, with a symptom relief rate of 21% (Figure 2).

According to the Nakai score, 11 (12.7%) cases had achieved excellent surgical outcomes, while good, fair, and poor were 60 (69.8%), 12 (14.0%), and 3 (3.5%), respectively. Patients were divided into two groups based on the Nakai score, which were the relief group with excellent or good in the Nakai score and the less relief group with fair or poor in the Nakai score. There were 71 cases in the relief group and 15 cases in the less relief group.

Demographics, preoperative clinical data, and imaging grade stratified by surgical outcomes are shown in Table 4. All the predictive variables were first assessed individually, and the significance levels are also listed in Table 4. Gender (*p*=0.193), BMI (*p*=0.184), preoperative SBI pain score (*p*=0.002), preoperative SBI numbness score (*p*=0.08), preoperative SBI weakness score (*p*=0.186), preoperative SBI total score (*p*=0.05), and preoperative ODI score (*p*=0.007) were included as covariates in the final model of surgical outcomes. Among them, preoperative SBI pain score (odds ratio [OR] = 1.647; 95% confidence interval [CI], 1.199–2.261; *p*=0.002) significantly increased the probability of relatively good surgical outcomes, while gender (*p*=0.242), BMI (*p*=0.175), preoperative SBI numbness score (*p*=0.302), preoperative SBI weakness score (*p*=0.126), preoperative SBI total score (*p*=0.525), and preoperative ODI score (*p*=0.059) were found to be nonsignificant predictors of the relatively good surgical outcomes and were excluded from the model.

To further explore the risk factors for preoperative presence of numbness and/or weakness, we divided the patients into the numbness and/or weakness group and the pain alone group based on the SBI subitem scores. Patients with SBI numbness and/or weakness scores greater than 2 points were grouped into the numbness and/or weakness group while the rest were in the pain alone group. Demographics, preoperative ODI score, duration of preoperative symptoms, and imaging grade stratified by presence of numbness and/or weakness are shown in Table 5. All the above predictive variables were first assessed individually, and the significance levels are also listed in Table 5. The reason for analyzing the multiplicativity of duration of preoperative symptoms and imaging grade was that they had complex interactions between them which might have influence on each other. Duration of preoperative symptoms (*p*=0.006), imaging grade (*p*=0.123), and joint effect of duration of preoperative symptoms and imaging grade (multiplicativity) (*p*=0.004) were included as covariates in the final model of presence of numbness and/or weakness. Among them, joint effect of duration of preoperative symptoms and imaging grade (odds ratio [OR] = 1.015; 95% confidence interval [CI], 1.005–1.026; *p*=0.004) significantly increased the probability of presence of numbness and/or weakness, while duration of preoperative symptoms (*p*=0.633) and imaging grade (*p*=0.341)individually were found to be nonsignificant predictors and were excluded from the model.

## 4. Discussion

Patients with lumbar disc herniation usually present symptoms like pain, numbness, and weakness, which have a negative influence on their social functions [1, 16]. Our results showed that 75 (87.2%) out of 86 patients had pain, 61 patients (70.9%) had numbness, and only 37 patients (43%) had weakness. Preoperatively, pain was the most common symptom, followed by numbness and weakness, which was consistent with the results of the previous literature [10]. Huang and Sengupta conducted a retrospective study in which they followed up 85 patients who had surgical decompression of the nerve root due to lumbar disease and they found that most of the patients had pain and numbness before surgery. Our results also showed that the preoperative pain score and weakness score correlated with the ODI score, while numbness did not, which indicated that although commonly presented in LDH patients, numbness did not have so much negative influence on their social function as pain and weakness did. This was consistent with our clinical observation. Numbness appeared to be more bearable than pain and caused less social dysfunction.

A great number of studies in the previous literature have reported significant relief of symptoms after decompression surgery [17–20]. Endoscopic procedures for treatment of spinal diseases firstly came out in 1992 and have been widely applied in clinical practice for lumbar spine diseases, as the rapid development of endoscopic technology. Recent studies have reported that they could achieve equal curative effect with less trauma and shorter hospital stay compared to traditional surgeries [3–6]. Our results showed that 71 (82.6%) out of 86 cases achieved good or excellent surgical outcomes which further confirmed that PELD was an effective and reliable method for the treatment of LDH.

However, previous studies mainly provided evidence about patients' postoperative overall functional recovery by employing outcome measurements like JOA, VAS, ODI, or SF36 [7–9]. Various rate and extent of symptoms relief had rarely been discussed. In Huang's research, pain recovered the fastest at the first 6 weeks after surgical decompression, while numbness recovered at a slower pace [10]. We obtained similar results in our study. At 3 months follow-up after PELD procedures, 82% of pain, 41% of numbness, and 21% of weakness were relieved compared with the preoperative symptoms. Pain relieved by the largest extent, which we inferred might be explained from the nerve fiber anatomic point of view. The spinal nerve consists of somatic sensory and motor nerve fibers with various diameters [21]. Damage to sensory A*β* fibers conducting feeling vibrations and touch may result in a general sense of numbness [22, 23], and damage to motor A*α* fibers may result in weakness [24, 25]. Both kinds of fibers are myelinated with relatively larger diameters. Pain on the other hand is conducted mainly by unmyelinated thin C fibers and partly by myelinated fibers [26]. Nerve root compressed by LDH would lead to impairment of intraneural microcirculation and tissue inflammatory process, and long-term compression might further induce damage and demyelination of nerve fibers. Decompression by PELD could recover the blood infusion and create a more beneficial environment for nerve fiber regeneration. C fibers recover more quickly and easily than A*α* and A*β* fibers as the process of remyelination requires more time [10].

The SBI scores have been used in the previous studies about lumbar disc herniation and have demonstrated acceptable reliability, construct validity, and responsiveness [11, 13]. Three of them were also incorporated in the North American Spine Society questionnaire [12]. Our results showed that the preoperative SBI pain score significantly increased the probability of relatively good surgical outcomes, which indicated that pain was the most significant outcome criteria for patients during the early stage after surgery. Patients with more preoperative pain tended to have more relief of symptoms postoperatively. This was consistent with our common clinical observation that pain relief contributed more to the overall symptoms relief than other symptoms like numbness and weakness, which was also consistent with our previous results that pain relieved by the largest extent. Theoretically, as caused mainly by larger myelinated nerve fibers, numbness and weakness tended to be more difficult to recovery, resulting in a worse surgical outcome. However, given the fact that most LDH patients presented pain simultaneously with other symptoms, it was reasonable that their effects on the surgical outcomes were confounded by pain. While numbness and weakness were not independent risk factors, we believed the results were still meaningful in clinical application because persistent numbness and/or weakness after satisfactory pain relief is commonly seen in patients during the postoperative follow-up [10]. By analyzing the risk factors for presence of numbness and/or weakness before surgery, we found that the joint effect of duration of preoperative symptoms and imaging grade significantly increased the probability of presence of numbness and/or weakness, and the interaction between them was multiplicative, which indicated that preoperative long duration of symptoms together with a severe compression of nerve root would lead to numbness and/or weakness. Long duration of symptoms and severe nerve root compression may induce deformation and demyelination of nerve fibers distally resulting in numbness and weakness.

This study has some limitations. The reason for following up patients at 3 months after operation was that most symptoms of LDH recovered the fastest at the first 6 weeks and plateaued at 3 months postoperatively according to the previous literature [10]. The results of our study might uncover the extent of symptoms relief at the early stage after operation. Yet, there was still slow improvement of these symptoms even until 1 year postoperatively. Further studies might be needed with a longer follow-up to reveal the overall relief extent of various symptoms. And the other limitation is that this study is a retrospective analysis of prospectively collected data, which may have potential biases of a retrospective study.

## 5. Conclusion

In patients with LDH, pain was the most common symptom, followed by numbness and weakness. PELD had a good curative effect in the treatment of LDH. Patients recovered best from pain, followed by numbness and weakness. Higher level of patients' self-reported preoperative pain indicated a better surgical outcome for LDH patients, and preoperative long duration of symptoms together with a severe compression of nerve root significantly increased the risk of presenting numbness and/or weakness.

## Figures and Tables

**Table 1:** Nakai score.

Scoring used in the study	
The patient has resumed work-related and other activities with slight or no symptoms	Excellent
The patient has resumed work-related and other activities but occasionally feels pain in the back or lower limbs after strenuous work	Good
The patient has reduced work-related and other activities because of residual pain in the back or lower limbs	Fair
The patient cannot work or carry out activities of daily living and is considered to be disabled	Poor

**Table 2:** Demographic, clinical, and imaging characteristics of enrolled patients.

Demographic data	
Age (years)	49 (21–80)
Gender (*n*, %)	
Male	44 (51%)
Female	42 (49%)
BMI (kg/m^2^)	25.2 (3.80)
Clinical data	
Duration of symptoms (weeks)	118 (1–1440)
Preop ODI	49 (22)
Preop SBI total	9.4 (4.35)
Preop SBI pain	3.7 (1.74)
Preop SBI numbness	2.9 (2.08)
Preop SBI weakness	0.7 (1.11)
Imaging grade	
A	32 (37.2%)
B	19 (22%)
C	35 (40.6%)

**Table 3:** Correlations between the preoperative ODI score and the SBI total score and its subitem scores.

Variables	ODI	
	Correlation coefficient	*p* value
SBI total	0.654	<0.001^*∗*^
SBI pain	0.552	<0.001^*∗*^
SBI numbness	0.148	0.173
SBI weakness	0.241	0.026^*∗*^

*p* value less than 0.05 was considered significant.

**Table 4:** Demographics, preoperative clinical data, and imaging grade stratified by surgical outcomes.

Variables	Relief group (71)	Less relief group (15)	*p* value^#^
Age, mean (SD)	48.9 (16.47)	51.6 (17.61)	0.572
Male, *n* (%)	34 (47.9)	10 (66.6)	0.193^#^
BMI, mean (SD)	25.4 (3.95)	24.0 (2.80)	0.184^#^
Duration of symptoms, mean (SD)	110 (246.5)	158 (253.72)	0.495
Preop ODI, mean (SD)	52.6 (22)	34.3 (20)	0.007^#^
Preop SBI total, mean (SD)	9.9 (4.55)	7.4 (2.41)	0.052^#^
Preop SBI pain, mean (SD)	4.0 (1.56)	2.3 (1.95)	0.002^#^
Preop SBI numbness, mean (SD)	2.7 (2.14)	3.7 (1.49)	0.08^#^
Preop SBI weakness, mean (SD)	0.6 (1.11)	1.1 (1.10)	0.186^#^
Imaging grade A, *n* (%)	29 (40.8%)	3 (20%)	0.267

For qualitative variables, the total number of patients and the percentage are provided. For quantitative variables, the mean and standard deviation are provided. These predictive variables are first assessed individually; the significance levels are also listed. ^#^Indicates predictive variables included in the final model.

**Table 5:** Demographics, preoperative ODI score, duration of preoperative symptoms, and imaging grade stratified by presence of numbness and/or weakness.

Variables	Numbness and/or weakness group (57)	Pain alone group (29)	*p* value^#^
Age, mean (SD)	50 (16.0)	48 (17.9)	0.486
Male, *n* (%)	31 (54.3)	13 (44.8)	0.403
BMI, mean (SD)	25.3 (4.08)	24.9 (3.24)	0.690
Duration of symptoms, mean (SD)	171 (290.3)	16 (16.0)	0.006^#^
Preop ODI, mean (SD)	51.3 (23.0)	45.6 (20.0)	0.266
Imaging grade A, *n* (%)	18 (31.5%)	14 (48.2%)	0.123^#^
Joint effect of duration of symptoms and imaging grade	—	—	0.004^#^

For qualitative variables, the total number of patients and the percentage are provided. For quantitative variables, the mean and standard deviation are provided. These predictive variables are first assessed individually; the significance levels are also listed. ^#^Indicates predictive variables included in the final model.

## Data Availability

The data used to support the findings of this study are available from the corresponding authors upon request.
